# Cell death and morphogenesis during early mouse development: Are they interconnected?

**DOI:** 10.1002/bies.201400147

**Published:** 2015-01-15

**Authors:** Ivan Bedzhov, Magdalena Zernicka-Goetz

**Affiliations:** 1) Department of Physiology, Development and Neuroscience, University of CambridgeCambridge, UK; 2) Wellcome Trust/Cancer Research UK Gurdon Institute, University of CambridgeCambridge, UK

**Keywords:** apoptosis, blastocyst, egg cylinder, epiblast, implantation, morphogenesis

## Abstract

Shortly after implantation the embryonic lineage transforms from a coherent ball of cells into polarized cup shaped epithelium. Recently we elucidated a previously unknown apoptosis-independent morphogenic event that reorganizes the pluripotent lineage. Polarization cues from the surrounding basement membrane rearrange the epiblast into a polarized rosette-like structure, where subsequently a central lumen is established. Thus, we provided a new model revising the current concept of apoptosis-dependent epiblast morphogenesis. Cell death however has to be tightly regulated during embryogenesis to ensure developmental success. Here, we follow the stages of early mouse development and take a glimpse at the critical signaling and morphogenic events that determine cells destiny and reshape the embryonic lineage.

## Introduction

**Figure 1:**
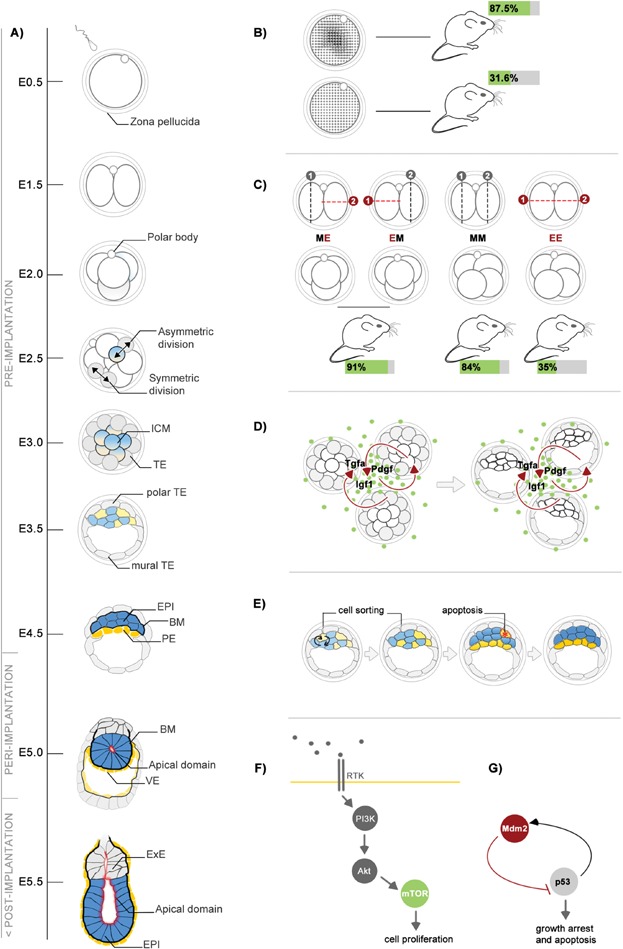
Determinants of embryo viability during early mouse development. A: Overview of the pre-, peri- and the early post-implantation stages of mouse embryogenesis. B: Patterns of cytoplasmic movements predict the developmental potential of the zygote. C: Cell division patterns of 2- to 4-cell stage transition in relation to the developmental success to term. D: Paracrine and autocrine pro-survival factors during pre-implantation embryogenesis. E: Cell sorting and elimination of mis-positioned cells by apoptosis during the second cell lineage segregation. F: Activation of the mTOR pathway downstream of the PI3K/Akt signalling cascade. G: Mdm2–p53 regulatory loop regulating embryo survival during the peri-implantation stages of development.

Sperm entry triggers the “big bang” that transforms a fertilized egg into a new organism. Cell fate choices generate a diversity of tissues that undergo morphogenetic transformations, reshaping the developing embryo. The first two cell fate decisions set up the embryonic and extraembryonic lineages. The embryonic lineage or the epiblast (EPI) contains pluripotent progenitors that give rise to all tissues of the foetus. The signals that organise and support the development of the EPI are provided by derivatives of the two extraembryonic lineages - the trophectoderm (TE) and the primitive endoderm (PE) (Fig. 1A). The fitness of these early lineages is essential for embryo survival and development to term. Cells that fail to segregate into appropriate positions according to their cell fate program, or lack survival signals, have to be eliminated to maintain tissue integrity and function. Cell death also directs morphogenic processes such as the sculpting of the digits of the vertebrate limb [1] and until recently was considered responsible for the establishment of the hollow tube of the egg cylinder [2,3]. Alternatively, central luminal space can be formed in a solid cell cluster by apoptosis-independent mechanism if strong polarization cues are provided [4,5]. In early embryos such signals originate from the basement membrane that surrounds the epiblast, and they drive polarization and lumenogenesis in the embryonic lineage during the peri-implantation stages [6]. This concept is contrary to the apoptosis-dependent mechanism of the textbook model describing epiblast morphogenesis from pre- to post-implantation stages [2,3].

## Apoptosis and necrosis - major mechanisms of cell death

Multiple cell generations maintain and expand the embryonic and extraembryonic structures throughout embryogenesis. This continuity relies on the tightly balanced processes of cell death and survival. Cell death can occur following the paths of necrosis or apoptosis. Necrosis is not a regulated process and is usually a result of physical damage or ischemia that compromises cell integrity leading to the release of cell contents into the external environment. In contrast, programmed cell death (PCD) or apoptosis is a multilevel, heavily regulated process, activated by external or cell intrinsic stimuli. The intrinsic apoptotic program can be triggered as a response to DNA damage, high calcium and oxidant levels or lack of survival signals, whereas the extrinsic apoptotic cascade is downstream of ligand activated cell surface receptors. Membrane blebbing, nuclear condensation and formation of apoptotic bodies are the typical morphological characteristic of cells undergoing apoptosis. Subsequently, the dying cells and debris are rapidly cleared from the tissue by efferocytosis [7,8].

## How are the processes of cell death and survival balanced during pre-implantation development?

Apoptosis is suggested as a default cell destiny that has to be continuously suppressed by survival signals [9]. Such signals can be provided via integrin-extracellular matrix interactions [10], cadherin mediated cell-cell contacts [11] or by soluble factors. Thus, a crosstalk of multiple signalling pathways tightly regulates the balance between cell death and survival. Embryo viability directly depends on the fitness of the zygote and embryo's capacity for full development to term can be predicted as early as the first hours post fertilization (Box 1). Observations of embryonic development in vitro show that embryos cultured in larger groups or in smaller volumes of medium develop to the blastocyst stage with higher rates and contain fewer cells undergoing apoptosis in comparison to individually cultured embryos [12,13]. The commonly used media are relatively simple, chemically defined solutions, containing no additional growth factors. Thus, embryos themselves produce soluble ligands acting in auto- and paracrine manner, and in larger volumes these factors are diluted out. Ligands, receptors and downstream signaling components of the Igf, Egf, Tgf-β and Pdgf families are expressed throughout pre-implantation development [14] (Fig. 1D). In addition, E-cadherin (E-cad) mediated adhesion, which is essential for proper blastocyst formation, also provides pro-survival cues. The E-cad extracellular domain interacts with Igf1r, mediating efficient activation of the receptor [15,16]. In turn, Igf1r triggers anti-apoptotic, metabolic and mitogenic responses in developing embryo through the PI3K/Akt pathway [17,18]. Activated Akt phosphorylates and sequesters pro-apoptotic factors such as BAD, thus keeping the intrinsic apoptotic program at bay [19].

The observed incidence of apoptosis at the blastocyst stage differs between the TE and the inner cell mass (ICM). PCD in the TE is a relatively rare event, whereas the rates of apoptosis in the ICM are significantly higher [20,21]. It is proposed that the apoptotic process in the ICM eliminates cells that failed to translocate to the correct position for their fate during the segregation of the PE and EPI cell populations [22–24] (Fig. 1E). However, the mechanism by which cells sense their “incorrect” position remains unknown. A potential autocrine loop of Egfr activation by the Tgf-α ligand is proposed to modulate the levels of PCD in the ICM [25]. This is also indicated by the depletion of Egfr, which results in complete ICM degeneration in the CF-1 mouse genetic background [26]. Maintenance of the PE depends on pro-survival cues downstream of Pdgfrα. Genetic ablation or pharmacological inhibition of this receptor result in increased Caspase-3 activity and depletion of the PE layer [27].

After the second lineage segregation is complete, the mature blastocyst hatches out of the zona and initiates implantation. The mural TE mediates the first interactions of the implanting blastocyst with the maternal environment. The direct contact between the TE cells and the uterine epithelium induces apoptosis at the attachment site, allowing penetration of the embryo into the underlying stroma. This apoptotic process is suggested to be a result of TNF-receptor I activation that triggers Caspase-3 mediated local PCD of the luminal epithelium [28,29].

## Multiple signaling pathways regulate apoptosis and survival during the peri-implantation and early post-implantation stages

As the embryo invades the maternal environment the surrounding stroma proliferates and transforms into the decidua that supports the growth of the developing egg cylinder and ensures foetomaternal immune tolerance [30]. Following implantation, multiple signaling cascades regulate the processes of cell death, survival and proliferation in the embryo. The Igf pathway promotes survival and cell proliferation by activating mTOR downstream of the PI3K/Akt cascade [31] (Fig. 1F). Genetic inactivation of class IA or class 3 PI3K results in embryonic lethality at the time or shortly after implantation [32,33]. Interestingly, inactivation of genes associated with neoplastic transformations in adult tissues, such as Brca1 and 2, lead to growth arrest of the early egg cylinders [34–36]. Another example is the loss of function of Mdm2 oncogene that results in a complete elimination of all cells in the embryo shortly after implantation. Mdm2 binds directly to p53 and inhibits the expression of p53 target genes (Fig. 1G). In the absence of Mdm2, p53 activity is not regulated and the intrinsic apoptotic program is triggered, killing the embryo by E5.5. The Mdm2–p53 regulatory circuit can be bypassed by combined deletion of both genes, rescuing the double knockout embryos [37,38]. Deletion of p53 alone does not affect embryonic development in general, although a subset of later (E13.5–E16.5) embryos exhibit exencephaly (location of the brain outside the cranial cavity) [39]. Thus, hyperactivation of p53 leads to global PCD and peri-implantation lethality, whereas inactivation of p53-dependent apoptosis has no effect on early embryogenesis.

## Does peri-implantation morphogenesis depend on the process of apoptosis or is there an alternative mechanism?

Following implantation the polar TE forms the extraembryonic ectoderm (ExE) at the proximal region of the egg cylinder. The ExE contains the multipotent progenitors of the trophoblast lineage that form the embryonic portion of the placenta. The PE layer differentiates into parietal endoderm (PE) that migrates over the mural TE surface and visceral endoderm (VE) that engulfs the developing egg cylinder. Signalling centers of the VE pattern the underlying EPI, breaking its symmetry to establish the anterior – posterior axis [40–43].

The process of EPI re-organization during peri-implantation stages has been a long-standing mystery. Embryos at those stages are no longer free-floating and as they invade the maternal tissues they become relatively inaccessible. The time of implantation is one of the most critical periods of development. Only embryos that successfully attach and implant stand a chance of completing embryogenesis. The process of implantation also boosts cell proliferation and, for the first time, embryo growth is initiated. As the egg cylinder emerges the EPI dramatically changes its morphology from a simple ball of unpolarised cells into a cup-shaped pseudostratified epithelium, surrounding the proamniotic cavity.

Cavitation and hollowing are the two major paths through which a solid cohort of cells can be transformed into a tube by the generation of a central luminal space. Cavitation is an apoptosis-dependent mechanism of cell elimination in the core of a coherent mass of cells. Hollowing is apoptosis-independent, but requires separation of apical membranes in a radially polarized structure [4,5]. In vitro, the same type of cells are able to follow either of these alternative paths, depending on cell density and the efficiency of establishing apical-basal polarity. For example, MDCK cells grown at low density and receiving strong polarization cues polarize rapidly and form a central lumen via hollowing. However, when MDCK cells are grown at high density, in the absence of strong polarization signals, the central space is gradually formed by apoptosis [44]. Which mechanism is utilized by the embryo to establish the cup-shaped EPI after implantation?

**Figure 2:**
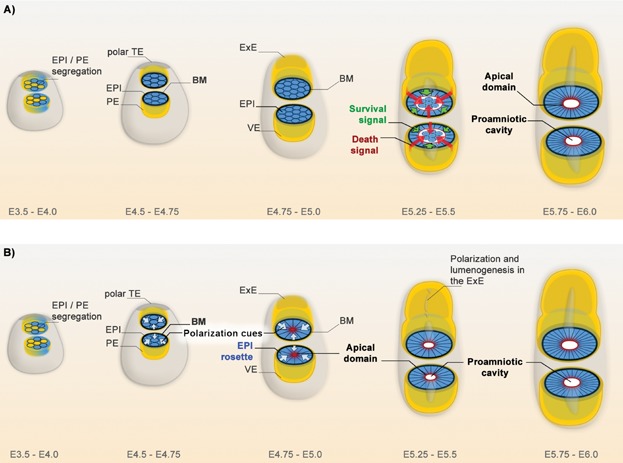
Models of peri-implantation morphogenesis. A: Apoptosis-dependent two-step signalling mechanism of cavitation in the early embryo. B: Self-organizing mechanism of EPI re-organization into a rosette-like structure, orchestrated by BM-provided polarization cues.

According to a long-standing simple and elegant two-step model, the EPI was thought to be reshaped by an apoptosis driven process of cavitation (Fig. 2A). This model proposes that at E5.0 the EPI of the early egg cylinder is a coherent mass of pluripotent cells. As the egg cylinder elongates, the VE provides an apoptotic signal to eliminate the cells in the core. The EPI cells in direct contact with the surrounding basement membrane (BM) are rescued and form polarized epithelium at the same time as the cavity is established [2,3]. The evidence supporting this model comes from studies using embryoid bodies (EBs) composed of embryonic stem (ES) or embryonic carcinoma (EC) cells. Cavitation in EBs never starts from the center, instead multiple peripheral cavities are established that coalesce as the core is gradually eliminated by PCD. EBs contain hundreds to thousands of cells and the processes of cavity formation and establishment of epithelial polarity are slow, over the course of several days [2,45]. Does the embryo follow the same path of EPI re-organization?

Using embryos directly isolated from the uterus or blastocysts cultured in vitro throughout the corresponding peri-implantation stages we revealed a strikingly different sequence of events (Fig. 2B). The polar TE and the PE secrete ECM proteins that establish a basement membrane (BM) that wraps around the EPI of the late E4.5 blastocyst [46,47]. Within 24 hours, polarization cues provided by the BM establish apical basal polarity in the EPI cells, through integrin mediated signalling. As a result, at the time of implantation the EPI globally re-organizes into a radially polarized rosette-like structure. Constriction of the actomyosin network reshapes the initially round cells, as the apical domains cluster in the center. Although apoptotic cells and cell debris can be found in some embryos, a single central lumen emerges independently of PCD through hollowing, most likely via charge repulsion of apical membranes. Fluid filling mechanisms such as exocytosis and pumping are likely to contribute to further enlarging the lumen. Similar morphogenic changes occur in the ExE, where the processes of polarization and lumenogenesis generate small intermembranous spaces that later contribute to the mature proamniotic cavity. During the differentiation of the polar TE into ExE, the BM that separates this lineage from the EPI is no longer maintained (Fig. 1A). Thus, at the early egg cylinder stage a common BM provided by the VE surrounds both the EPI and the ExE. Since the EPI cells are anchored to the ECM by integrins, the basket-shaped BM can act as a mold, transforming the symmetric rosette into a cup [6,48].

The BM function in the establishment of apical basal polarity can be substituted in vitro by culturing isolated ICMs in 3D ECM. The EPI cells polarize and initiate lumenogenesis in the absence of a surrounding VE layer, indicating that a death signal from the VE is not required for this process. Similar morphogenetic changes can be induced in ES cells when they are embedded into 3D ECM at low density. The ES cell spheres formed in these conditions efficiently polarize and establish central lumen, mimicking the pre- to post-implantation transition of the EPI lineage [6]. Together these observations lead to a new model of peri-implantation morphogenesis, where the BM established by the extraembryonic tissues acts as niche providing instructive signals for the self-organization of the EPI independently of apoptosis.

## Conclusions and outlook

Cytoplasmic movements in the zygote and cell division patterns of the 2-cell stage embryo are the earliest indicators of an embryo's potential for full development to term. As cleavage divisions progressively generate smaller blastomeres, multiple paracrine and autocrine signals promote embryo viability. The balance between cell survival and apoptosis is tightly regulated to maintain the functional integrity of the early lineages. A burst of cell proliferation in the implanting embryo expands all cell lineages, alongside a dramatic morphogenetic transformation of the EPI. Cells that are damaged or miss-positioned are most likely eliminated through apoptosis; however the process of PCD is dispensable for the morphogenetic changes that take place at that time. The BM components secreted by the extraembryonic lineages establish a niche that provides polarization cues to the pluripotent cells of the implanting embryo. In line with this, embryos that fail to assemble BM or lack β1-integrin receptors die during the implantation stages [47,49,50]. Similarly, genetic ablation of ILK and Pinch1 adaptor proteins result in peri-implantation lethality [51–53]. Thus, the integrin adaptor complex can serve as a mediator, transmitting signals for polarization in the EPI from the surrounding BM. The small GTPase Cdc42 appears as a central player in this process. In epithelial cells Cdc42 regulates actin polymerisation, cell junctions assembly and signals to the myosin light-chain kinase and the apical determinants of the Par complex [54]. During early embryogenesis Cdc42 is indispensable for the establishment of epithelial polarity in the post-implantation EPI [55,56]. A final question remains: what is the exact signalling that consolidates signals from the BM and central epithelial factors such as Cdc42 to establish apical-basal polarity in the embryonic lineage, and how this process is developmentally regulated remains to be determined.

**Box 1 How can early embryo survival be predicted?**

PCD is involved in the process of oogenesis, where most of the oocytes are lost during the first stages of meiotic prophase I [57]. Thus, the females are born with a substantially lower number of oocytes in comparison to the early stages of oogenesis. The mature oocytes can activate multiple signaling cascades, driving cell death through apoptosis, necrosis and autophagy [58]. After fertilization the first typical features of PCD can be observed in the polar bodies [59]. Since embryogenesis directly depends on the fitness of the zygote, can the potential for full development be predicted at that stage? Fertilization of the egg triggers cytoplasmic Ca^2+^ oscillations that induce contractions of the actomyosin network, manifested as rhythmic cytoplasmic movements. Using high-speed time-lapse imaging the patterns of cytoplasmic movements have recently been related to the developmental potential of the zygote. Frequent speed peaks, as well as a low mean basal speed of cytoplasmic movement, were found to be associated with a low rate of development to the blastocyst stage in vitro and to birth in vivo (Fig. 1B). Thus, the dynamics of cytoplasmic flows triggered by the sperm are predictive of the embryo's potential to develop to term [60].

The regulative development of pre-implantation embryos enables adaptation to reductions in cell number. If one of the 2-cell stage blastomeres is destroyed, an individual totipotent blastomere can still complete embryonic development [61]. Within one round of cell divisions, from the 2- to 4- cell stage, single blastomeres lose capacity for full development to term [62]. How can this be explained? On the one hand the 4-cell stage blastomeres are smaller, since the cleavage divisions during the pre-implantation period increase the cell number, but the total cell volume remains constant. These individual blastomeres are still able to form small blastocyst-like structures, known as trophoblastic vesicles, but they fail to develop further. This could be because a minimum of four EPI cells needs to be present in the blastocyst before implantation for successful establishment of the embryo proper, and this number is never reached in embryos derived from individual 4-cell stage blastomeres [63]. On the other hand, the cleavage pattern of the 2- to 4-cell stage transition was also found to influence developmental success. The zygote and 2-cell stage blastomeres can divide either meridionally (M) or equatorially (E). These sequential divisions generate four different kinds of embryos: ME, EM, MM or EE (Fig. 1C). In contrast to the other three classes, the development of EE embryos to term is significantly reduced [64]. To elucidate the underlying molecular mechanism and potentially identify a unique EE transcriptional signature, it will be important to correlate the transcriptional profile of individual 4-cell stage blastomeres with their division pattern. It also remains to be determined whether the history of the EE divisions can be traced back and correlated with the cytoplasmic movements of zygotes that have predicted reduced developmental potential.

## Abbreviations

BM: basement membrane
EB: embryoid body
EC cell: embryonic carcinoma cell
ECM: extracellular matrix
EPI: epiblast
**ES** cell: embryonic stem cell
ExE: extraembryonic ectoderm
ICM: inner cell mass
PCD: programmed cell death
PE: primitive endoderm
TE: trophectoderm
VE: visceral endoderm
